# The relationship between social support and health-promoting lifestyles among perimenopausal women: the mediating role of psychological resilience

**DOI:** 10.1186/s12905-025-04258-1

**Published:** 2026-01-22

**Authors:** Jie Lu, Yuan Yuan, Shiyu Wei, Kangfen Li, Suhua Tu, Lingling Xie

**Affiliations:** 1 Emergency Intensive Care Unit, The Affiliated Hospital of Southwest Medical University, Sichuan, China; 2 School of Nursing, Southwest Medical University, Sichuan, China; 3 The Second Affiliated Hospital of Chongqing Medical University, Chongqing, China; 4 Nursing Department, The Affiliated Hospital of Southwest Medical University, Sichuan, China; 5 Department of Gynaecology, The Affiliated Hospital of Southwest Medical University, Sichuan, China

**Keywords:** Perimenopause, Health-promoting lifestyle, Social support, Psychological resilience

## Abstract

**Background:**

Perimenopause presents considerable physical and psychological challenges. Adopting a healthy lifestyle is crucial for navigating this transition. While social support and psychological resilience are known to be related to health behaviours, their interrelationships among perimenopausal women remain unclear.

**Methods:**

This cross-sectional study enrolled 366 perimenopausal women from a health management centre in Southwest China between February and June 2023 using a convenience sampling method. Data were collected via a structured questionnaire comprising several sections: the demographic characteristics questionnaire, the Chinese version of the Health-Promoting Lifestyle Profile, the Social Support Rating Scale, and the Chinese version of the Connor-Davidson Resilience Scale. Following data entry in Excel 2016, statistical analyses, including descriptive statistics, correlation analysis, and multivariate regression, were conducted using SPSS 26.0. Structural equation modelling was performed with AMOS 24.0 to test the hypothesized pathways among variables. The significance level was set at α = 0.05, and model fit was evaluated using established goodness-of-fit indices.

**Results:**

The mean health-promoting lifestyle profile score was 98.01 (SD = 18.60). Multiple linear regression revealed several significant factors: menopausal status (*β* = 3.228, *P* = 0.033), the presence of chronic diseases (*β* = -4.761, *P* = 0.012), having regular medical examinations (*β* = 6.275, *P* < 0.001), social support (*β* = 0.358, *P* < 0.001), and psychological resilience (*β* = 0.567, *P* < 0.001). The SEM results further suggested that the association between social support and a healthy lifestyle was consistent with an intermediary role for psychological resilience. The indirect association was significant (95% bootstrap *CI*: 0.495–0.929), accounting for 61.32% of the total association.

**Conclusion:**

Social support is directly associated with healthier lifestyles among perimenopausal women, and psychological resilience appears to play an important intermediary role. Strengthening both social support and psychological resilience may help promote better health behaviours in this population. Health care professionals should consider integrating these elements into tailored perimenopausal health programs.

## Introduction

Perimenopause refers to the stage from the decline in ovarian function until one year after menopause. This period marks a physiological transition from reproductive age to older adulthood [1]. During this transition, women often experience a range of symptoms, including psychological and neurological symptoms, osteoporosis, menstrual irregularities, and increased cardiovascular risk [2, 3]. In addition to biological factors, sociocultural elements such as gender role expectations and social support systems profoundly influence health experiences [4]. Addressing health during perimenopause is crucial not only for women’s quality of life but also for broader familial and societal well-being [5]. With increasing life expectancy, the health management of the growing perimenopausal population is an important public health priority.

A health-promoting lifestyle (HPL) includes behaviours such as balanced nutrition, regular physical activity, stress management, and avoiding harmful habits. These activities help maintain physical and mental health while preventing disease [6, 7]. For perimenopausal women, adopting such a lifestyle is particularly important, as it can help alleviate menopausal symptoms and improve overall quality of life [8].

Psychological resilience—the capacity to adapt positively to stress—is another key resource during this life stage [9]. Hormonal fluctuations during perimenopause can contribute to mood disturbances, which may diminish resilience [10]. Greater resilience has been consistently associated with better health outcomes and more effective stress coping skills [11]. Specifically among perimenopausal women, greater resilience is linked to fewer depressive symptoms [12], lower severity of menopausal complaints [13], and higher overall life quality and satisfaction [14, 15].

In addition to individual resilience, social support plays a crucial role. Social support encompasses both material assistance from social networks and emotional encouragement, which together buffer stress and enhance coping capacity [16]. Adequate social support can mitigate negative emotions among perimenopausal women and help sustain mental well-being [17]. Social support is closely associated with an HPL and is a significant variable related to lifestyle outcomes [18]. Social support not only helps individuals cope with adversity by enhancing their psychological resilience but also increases their likelihood of adopting self-protective behaviours [19, 20].

Although the importance of social support is widely acknowledged, few studies have investigated how social support, psychological resilience, and HPLs interact specifically among perimenopausal women. This gap highlights the need for further investigation. Therefore, this cross-sectional study aimed to investigate the factors associated with HPLs among perimenopausal women and to examine the interrelationships—including the potential intermediary role of psychological resilience—between social support and lifestyle outcomes. The findings are intended to inform supportive strategies that enhance women’s active and healthy living during this transition.

### Theoretical framework and hypothesis formulation

The multipathway conceptual model of social support networks posits that social support influences health through several synergistic mechanisms [21]. Specifically, social support networks systematically affect individuals’ health behaviours, psychological states, and physiological functions by providing emotional and instrumental resources, transmitting social norms, facilitating social participation, and enhancing access to external resources. Existing research highlights health behaviour and psychological pathways as particularly salient in explaining how social support operates [22, 23]. Building on this model, the present study proposes that among perimenopausal women, social support may serve as a direct antecedent of both HPLs and psychological resilience.

Furthermore, the buffering hypothesis of social support suggests that social support mitigates the adverse health effects of stress through two key mechanisms: first, by attenuating threat appraisals during the cognitive evaluation of stressors, and second, by fostering adaptive coping responses during the stress reaction phase [24]. This process is theorized to strengthen key psychological resources—such as psychological resilience—which in turn promote engagement in health-protective behaviours. Perimenopause is a transitional period marked by physiological, psychological, and social changes that often increase stress and disrupt health-related practices. Within the buffering framework, social support may thus function both as a direct resource for health (main effect) and as a moderator of stress-related disturbances (buffering effect). Psychological resilience, defined as the capacity to adapt and thrive amid adversity, may be cultivated through social support and subsequently facilitate the maintenance of healthy behaviours [25]. On the basis of this reasoning, the present study proposes that psychological resilience mediates the relationship between social support and HPLs among perimenopausal women.

**Fig. 1:**
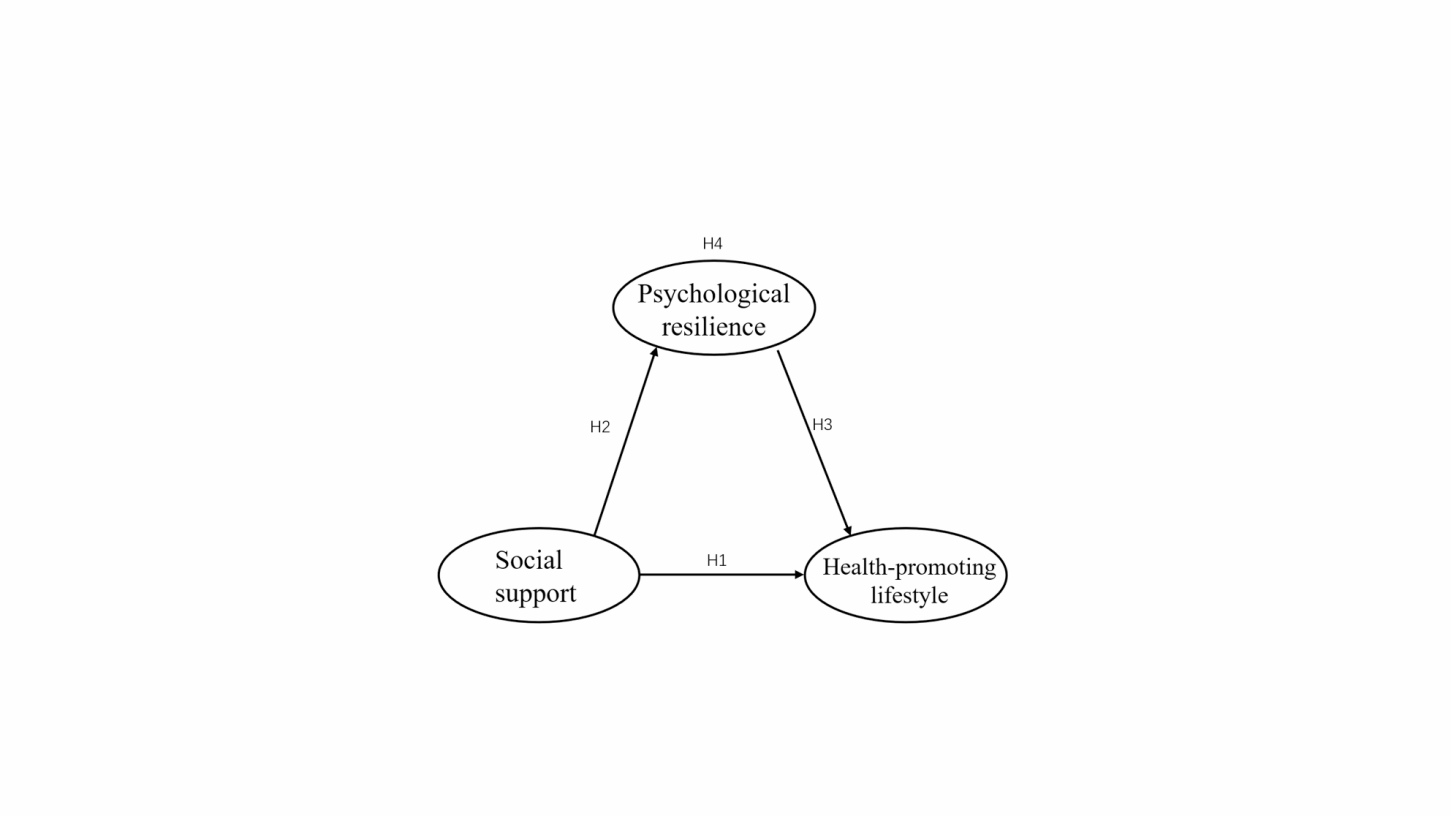
Theoretical framework

Integrating these perspectives, we propose a conceptual model that delineates the relationships among social support, psychological resilience, and HPLs in perimenopausal women. Specifically, we hypothesize that social support positively influences HPLs both directly and indirectly through enhanced psychological resilience. This model aligns with the buffering hypothesis and a socioecological understanding of health, illustrating how social resources bolster psychological adaptation, thereby encouraging the adoption of health-promoting behaviours during a critical life transition. As illustrated in Fig. 1, we propose the following hypotheses:

- H1: Social support is positively associated with an HPL.
- H2: Social support is positively associated with psychological resilience.
- H3: Psychological resilience is positively associated with an HPL.
- H4: Psychological resilience mediates the relationship between social support and an HPL.

## Methods

### Study design

This cross-sectional survey used convenience sampling. Perimenopausal women attending medical examinations at the Health Management Centre of the Affiliated Hospital of Southwest Medical University (Luzhou, Sichuan Province) between February and June 2023 were recruited.

### Inclusion and exclusion criteria

Eligible participants were perimenopausal women classified according to Stages of Reproductive Aging Workshop (STRAW) [1], with intact uterus and adnexa, able to communicate and cooperate, and who provided informed consent. Exclusion criteria included age under 40, serious organ diseases (e.g., renal failure, heart failure, malignancies), and incomplete questionnaires.

### Sample size

The required sample size was estimated on the basis of the guidelines recommending 10 observations per variable for multivariate analyses [26]. Given the 23 variables included in our initial analysis plan, a minimum of 230 participants were needed. To account for potential data attrition (e.g., invalid or incomplete questionnaires), this target was increased by 20%, resulting in a final minimum recruitment target of 276 participants. Our final sample of 366 perimenopausal women exceeded this target, providing adequate statistical power for the planned analyses, including multiple linear regression and structural equation modelling.

### Sampling procedures

Of 380 questionnaires distributed, 9 participants withdrew and 5 provided incomplete responses. After screening, 366 valid questionnaires were included for analysis.

### Measurement

(1) Demographic characteristics questionnaire: This questionnaire was designed to gather key sociodemographic and health-related factors. Prior literature has indicated that these factors could serve as correlates of health behaviours in perimenopausal women [8, 27]. Specifically, this questionnaire primarily collects basic demographic information about perimenopausal women, including age, literacy level, number of children, employment status, monthly income, place of residence, marital status, whether they undergo an annual medical examination, whether they live alone, their menopausal status, age at menopause, and whether they are afflicted with chronic diseases.

(2) Health-Promoting Lifestyle Profile (HPLP): The HPLP, developed by Walker et al. [28] and translated into Chinese by Cao [29], has been validated in Chinese populations with a Cronbach α of 0.91 and a split-half reliability of 0.92. The questionnaire consists of 40 items across six dimensions: interpersonal support, health responsibility, stress management, nutrition, physical activity, and self-actualization. Responses are rated on a 4-point Likert scale (1 = never, 4 = always), with total scores ranging from 40 to 160; higher scores indicate healthier lifestyles. The Chinese version of the HPLP-II demonstrated good internal consistency in our sample: overall scale (Cronbach α = 0.950) and for its subscales: interpersonal support (Cronbach α = 0.890), health responsibility (Cronbach α = 0.882), stress management (Cronbach α = 0.754), nutrition (Cronbach α = 0.836), physical activity (Cronbach α = 0.884), and self-actualization (Cronbach α = 0.898).

(3) Social Support Rating Scale (SSRS): This scale, widely utilized in Chinese populations, has demonstrated robust psychometric properties, with a reported Cronbach α of 0.896 and a test-retest reliability of 0.92 [30]. The SSRS comprises 10 items structured across three dimensions: subjective support, objective support, and social support utilization. Items 1–4 and 8–10 are rated on a 4-point Likert scale (1–4). Item 5 assesses the perceived level of family support and is scored from 1 (“none”) to 4 (“full support”). Items 6 and 7 evaluate the scope and sources of social support, with scores ranging from 0 to 9. The total score, obtained by summing the three subscale scores, ranges from 12 to 66, with higher scores indicating greater perceived social support. In the present study, the standardized Cronbach α coefficient for the SSRS was 0.805.

(4) Connor-Davidson Resilience Scale (CD-RISC) Inventory Chinese version:

The CD-RISC, originally developed by Connor and Davidson [31], has been widely translated and culturally adapted. Its Chinese version, translated and revised by Yu [32], has been validated in Chinese populations, with a Cronbach α of 0.91. This 25-item scale measures psychological resilience across three dimensions: tenacity (13 items), strength (8 items), and optimism (4 items). Responses are rated on a 5-point Likert scale (0 = never, 4 = always), with total scores ranging from 0 to 100; higher scores reflect greater psychological resilience. The standardized Cronbach α coefficient for the CD-RISC in the present study was 0.945.

### Investigation methods

Data were collected face-to-face at the health management centre while participants rested after their medical examinations. Researchers applied inclusion/exclusion criteria, explained the study purpose and procedures, and obtained informed consent before questionnaire completion.

### Statistical analysis

The data were analysed using SPSS 26.0 and AMOS 24.0. Descriptive statistics included frequencies, percentages, and means ± standard deviations. Pearson’s correlation, one-way ANOVA, independent samples t tests, and multiple linear regression were used to identify influencing factors. Structural equation modelling (SEM) with bootstrap testing was applied to examine pathways and mediation effects. Statistical significance was set at α = 0.05 (*P* < 0.05). Prior to testing the structural model, confirmatory factor analysis (CFA) was conducted to assess the measurement models for the latent constructs (social support, psychological resilience, and HPL). Structural equation modelling (SEM) was then performed using AMOS to examine the pathways among variables, with indirect associations tested via the bootstrap method. Model fit was assessed using the following criteria: a χ²/df < 3, a comparative fit index (CFI) > 0.90, a Tucker‒Lewis index (TLI) > 0.90, and a root mean square error of approximation (RMSEA) < 0.08. To control for potential confounding factors, relevant demographic and health-related covariates were included in the multivariable analyses. In the multiple linear regression, all covariates were entered simultaneously; in the SEM, they were specified as exogenous observed variables with direct paths to the outcome (HPL).

### Data quality control

Prior to data collection, key study variables were defined through extensive literature review. Researchers received standardized training to ensure consistency. Participants were informed of confidentiality principles and correct questionnaire procedures. During data entry, double-entry in Excel and consistency checks were performed to minimize errors.

### Ethics approval

This study was approved by the Clinical Trial Ethics Committee of the Affiliated Hospital of Southwest Medical University (Approval No.: KY2023023). Written informed consent was obtained from all participants. Participants were explicitly informed of their right to withdraw from the study at any time without penalty. To acknowledge their participation, a small gift was provided to each participant upon completion of the questionnaire.

## Results

### Demographic characteristics of survey participants and univariate analysis of HPL

A total of 366 perimenopausal women were included in the final analysis. The participants had a mean age of 47.58 years (SD = 3.53), and the mean age at menopause was 48.75 years (SD = 2.97). Significant differences in HPLP scores were observed across literacy level (*P* < 0.001), number of children (*P* = 0.003), employment status (*P* = 0.007), monthly income (*P* < 0.001), annual medical examination status (*P* < 0.001), menopausal status (*P* = 0.017), and the presence of chronic disease (*P* = 0.005). Specifically, higher literacy and undergoing annual physical examinations were associated with healthier lifestyle scores. Additionally, employed and retired participants had significantly higher scores than unemployed participants did. Postmenopausal women also had higher scores than their premenopausal counterparts did. Detailed information is presented in Table 1.

**Table 1:** Comparison of HPLP scores for participants with different characteristics

Variable	Score (Mean ± SD)	F/t	*P*	Pairwise Comparison
Age		1.601	0.203	
< 45 years old	100.51 ± 17.89			
45–49 years old	96.32 ± 18.47			
> 49 years old	99.15 ± 19.13			
Literacy Level		12.822	<0.001	①<②<③
①Elementary school and below	92.48 ± 17.84			
②Junior High School/High School	98.20 ± 17.49			
③University and above	105.26 ± 19.20			
Number of Children		5.853	0.003	②<①
①1 or below	101.29 ± 18.71			
②2	94.93 ± 17.91			
③3 or more	93.57 ± 18.64			
Employment Status		5.012	0.007	③<①,②
①Employed	99.62 ± 19.02			
②Retired	100.81 ± 16.03			
③Unemployed	92.70 ± 17.27			
Monthly Income		11.551	<0.001	①<②and ①,②,③<④
①0 RMB	90.95 ± 16.89			
②Under 2000RMB	97.30 ± 18.84			
③2000-5000RMB	96.29 ± 18.32			
④More than 5000RMB	108.76 ± 16.04			
Place of Residence		1.331	0.184	
Urban	99.15 ± 18.97			
Rural	96.55 ± 18.08			
Marital Status		0.719	0.472	
Unmarried/Divorced/Widowed	100.77 ± 17.91			
Married	97.83 ± 18.66			
Do you live alone?		-1.943	0.055	
Yes	94.42 ± 14.28			
No	98.67 ± 19.24			
Menopausal		2.398	0.017	
yes	100.74 ± 19.09			
No	96.04 ± 18.03			
Chronic Disease		-2.842	0.005	
yes	91.89 ± 17.00			
No	99.23 ± 18.69			
Annual Medical Examination		6.208	<0.001	
Yes	102.56 ± 18.26			
No	90.74 ± 16.81			

In the “Pairwise comparison” column, “① < ②” indicates that the mean score of group ① is significantly lower than that of group ② (*P* < 0.05)

In the “*F/t*” column, *F* values are reported for comparisons among more than two groups, while *t* values are reported for comparisons between two groups

### HPLP scores of survey participants

The mean score for HPLP in this study was 98.01 (SD ± 18.60). The item score (mean ± SD) is calculated by dividing the total/dimension score by the number of items in that scale or dimension, indicating the average response per dimension. As shown in Table 2, the mean scores were highest for the nutrition dimension and lowest for the physical activity dimension.

**Table 2:** HPLP and scores in each dimension

Category	Scoring Range	Total/Dimension Score (Mean ± SD)	Item Score (Mean ± SD)
HPLP	40–160	98.01 ± 18.60	2.45 ± 0.47
Interpersonal Support	5–20	13.29 ± 3.71	2.66 ± 0.74
Health Responsibility	11–44	24.01 ± 5.27	2.18 ± 0.48
Stress Management	5–20	12.59 ± 2.54	2.52 ± 0.51
Nutrition	6–24	18.19 ± 3.55	3.03 ± 0.59
Physical Activity	8–32	16.53 ± 4.80	2.07 ± 0.60
Self-Actualization	5–20	13.39 ± 3.90	2.68 ± 0.78

### Correlation analysis of the HPL, social support, and psychological resilience of survey participants

Correlation analysis revealed significant positive associations among HPL, social support, and psychological resilience (all *P* < 0.01). Please refer to Table 3 for detailed results.

**Table 3:** Correlation analysis of HPL, social support, and psychological resilience

Category	HPL	Social Support
Social Support	0.521^**^	1
Psychological Resilience	0.651^**^	0.556^**^

^**^ At the 0.01 level (two-tailed), *P* < 0.01

### A regression analysis of factors influencing the HPL of survey participants

**Fig. 2:**
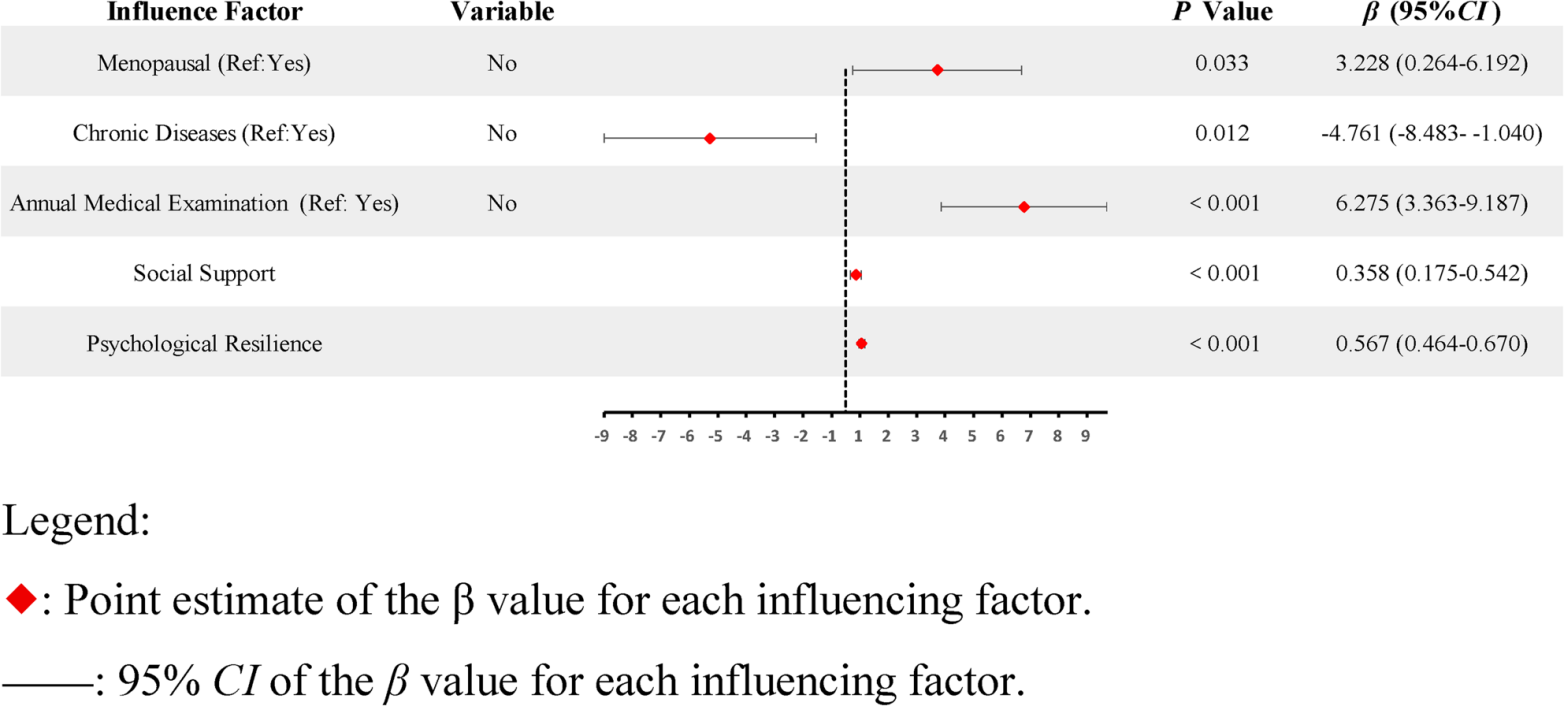
Forest plot of linear regression analysis of factors influencing HPL. Note: R2=0.529, adjusted R2=0.516, F=39.891, *P*<0.001

We used variables that were statistically significant in univariate and correlation analyses as independent variables. We used the total score of the HPLP as the dependent variable. We treated the categorical variables as dummy variables and then used them as independent variables in the regression equations. Regression analysis revealed menopause status, annual medical examinations, chronic disease status, social support, and psychological resilience as significant predictors of an HPL. Together, these variables explained 51.6% of the variance (adjusted *R²* = 0.516). The results of the regression analysis are summarized in Table 4. The forest plot of the regression coefficients is shown in Fig. 2.

**Table 4:** Linear regression analysis of factors influencing HPL

Variable	β	SE	95% CI	*P*
Menopausal (Ref: Yes)	3.23	1.507	[0.26, 6.19]	**0.033**
Chronic Disease (Ref: Yes)	-4.76	1.892	[-8.48, -1.04]	**0.012**
Annual Medical Examination (Ref: Yes)	6.28	1.48	[3.36, 9.19]	**< 0.001**
Social Support (continuous)	0.36	0.093	[0.18, 0.54]	**< 0.001**
Psychological Resilience (continuous)	0.57	0.052	[0.46, 0.67]	**< 0.001**
Literacy Level (continuous scoring)	1.6	1.154	[-0.67, 3.87]	0.167
Number of Children (continuous scoring)	-1.75	1.202	[-4.11, 0.62]	0.147
Employment Status: Employed (Ref: Retired)	-4.37	2.832	[-9.94, 1.20]	0.124
Employment Status: Employed(Ref: Unemployed)	-3.61	2.209	[-7.95, 0.74]	0.104
Monthly Income (continuous)	-0.01	1.128	[-2.23, 2.21]	0.992

Categorical variables (menopausal status, chronic disease status, annual medical examination attendance, and employment status) were dummy-coded, with the reference group indicated in parentheses. The ordinal variables, including literacy level (scored as follows: 1 = elementary school and below, 2 = junior high school/high school, and 3 = university and above), monthly income, and number of children, were treated as continuous in the regression model on the basis of their ordered scoring. Social support and psychological resilience were entered as continuous variables

### Suitability for factor analysis and common method bias

Prior to testing the structural model, we examined the suitability of the data for factor analysis. As shown in Table 5, the KMO values for all the scales and the overall model exceeded 0.7, and the results of Bartlett’s tests of sphericity were significant (*P* < 0.001), indicating that the data were appropriate for factor analysis. Furthermore, our sample size (*N* = 366) was adequate for SEM, with a ratio of observations to observed variables well above the recommended 10:1 threshold.

**Table 5:** Results of KMO and bartlett’s test for each scale

Scale	KMO Value	χ²	DF	*P*-value
SSRS	0.781	1193.62	45	< 0.001
CD-RISC	0.955	5246.66	300	< 0.001
HPLP	0.940	8219.40	780	< 0.001

KMO values range from 0 to 1, with values above 0.80 considered meritorious, above 0.70 considered middling, and above 0.60 considered mediocre but acceptable for factor analysis

### Assessment of multivariate normality and robust estimation

Prior to testing the hypothesized structural model, we examined the assumption of multivariate normality. The results of Mardia’s test indicated a significant departure from normality, with a critical ratio for multivariate kurtosis of 6.213 (*P* < 0.001). Therefore, to ensure robust parameter estimation, all subsequent structural equation modelling analyses were conducted using a bootstrap procedure with 5000 resamples [33]. Accordingly, path coefficients and mediation effects are reported with bias-corrected 95% confidence intervals derived from this bootstrap analysis, an approach that does not rely on distributional assumptions and provides more reliable inferences under conditions of multivariate nonnormality.

### Confirmatory factor analysis

The results of the CFA supported the validity of the measurement models. As shown in Table 6, all standardized factor loadings were above 0.60, composite reliability (CR) values exceeded 0.75, and average variance extracted (AVE) values were above 0.50, indicating adequate convergent validity and internal consistency for all latent constructs.

**Table 6:** Results of CFA for the measurement models

Construct	Indicator	factor loadings	SMC (*R*²)	CR	AVE
social support	subjective support	0.727	0.529	0.763	0.518
	objective support	0.724	0.524		
	social support utilisation	0.709	0.502		
psychological resilience	tenacity	0.859	0.738	0.896	0.743
	strength	0.944	0.893		
	optimism	0.773	0.598		
HPL	Interpersonal Support	0.763	0.583	0.861	0.509
	Health Responsibility	0.732	0.536		
	Stress Management	0.664	0.442		
	Nutrition	0.669	0.448		
	Physical Activity	0.668	0.447		
	Self-Actualization	0.773	0.598		

### Comparison of alternative models

To determine the most appropriate representation of the relationships among social support, psychological resilience, and HPL, we compared the fit of four theoretically derived structural models: (Model 1) a direct-effects model (social support → HPL), (Model 2) a partial mediation model (social support → HPL and social support → psychological resilience → HPL), (Model 3) a full mediation model (social support → psychological resilience → HPL), and (Model 4) a reversed unidirectional model (HPL → psychological resilience → social support). The fit indices for all the competing models are presented in Table 7. Nested model comparisons using chi-square difference tests were conducted to statistically evaluate the models. The partial mediation model (Model 2) demonstrated a significantly better fit than the direct effects model (Model 1), Δχ²(25) = 65.86, *P* < 0.001. These findings indicate that incorporating psychological resilience as a mediator significantly improves the model. Furthermore, the partial mediation model provided a significantly better fit than the more restrictive full mediation model did (Model 3), Δχ²(1) = 16.93, *P* < 0.001, indicating that the direct path from social support to HPL is statistically necessary and should not be omitted. Finally, compared with the reversed nonhypothesized model (Model 4), all the hypothesized models (1–3) fit the data substantially better, supporting our proposed directional relationships. On the basis of these results, the partial mediation model (Model 2) was retained as the final and best-fitting model.

**Table 7:** Fit indices for the competing structural models

Model	χ²	DF	χ²/DF	CFI	TLI	RMSEA [90% CI]	AIC	BIC
1	75.223	26	2.89	0.96	0.95	0.072 [0.053, 0.091]	113.223	187.37
2	141.083	51	2.77	0.96	0.95	0.070 [0.056, 0.083]	195.083	300.45
3	158.012	52	3.04	0.95	0.94	0.075 [0.062, 0.088]	210.012	311.481
4	197.395	52	3.80	0.94	0.92	0.088 [0.075, 0.101]	249.395	350.863

### Model construction and fit

**Fig. 3:**
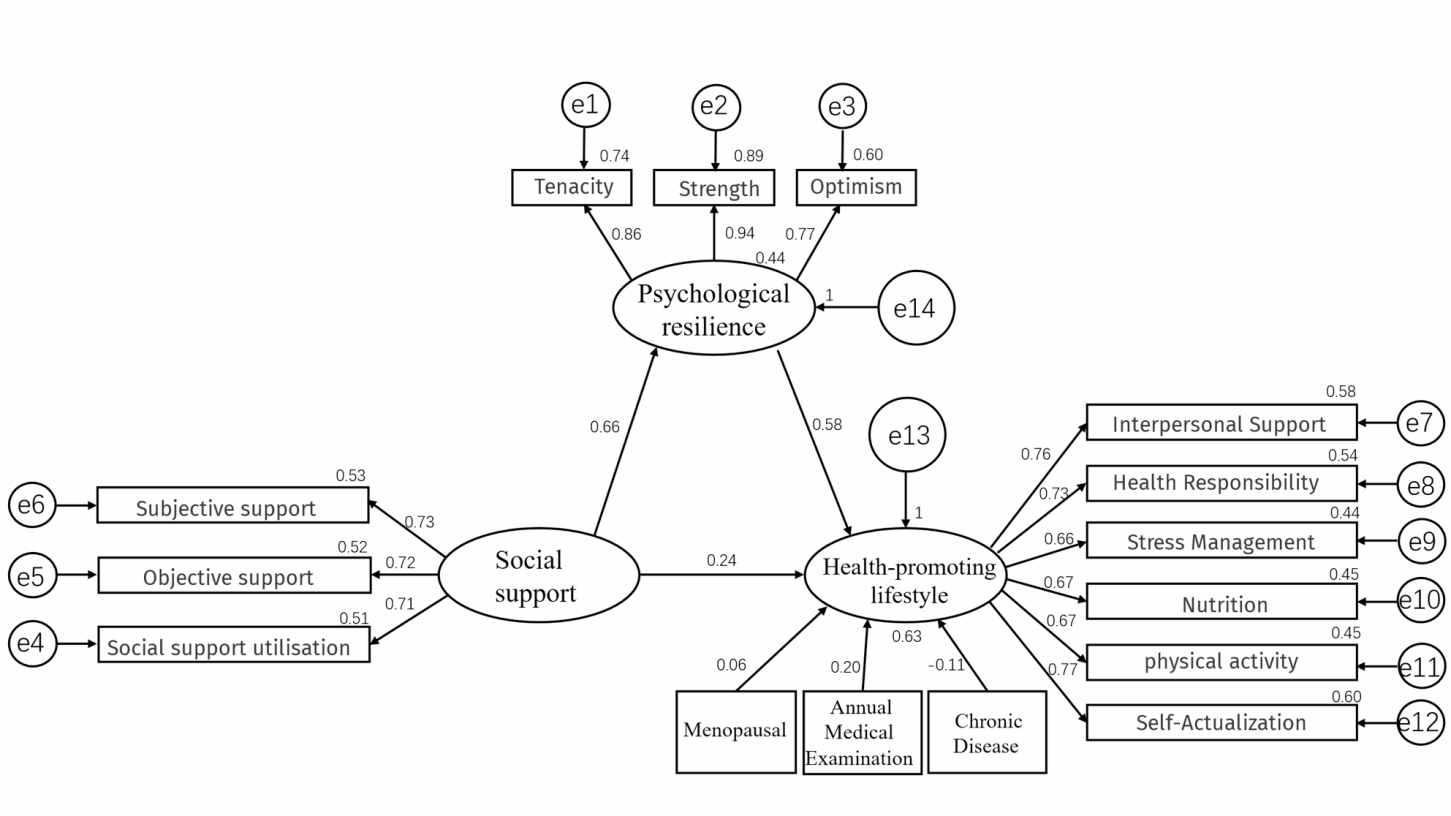
Fitted model diagram

Using AMOS 24.0, a structural equation model was constructed to examine the relationships among HPL, social support, and psychological resilience in perimenopausal women. HPL was specified as the dependent variable; social support as the independent variable; menopausal status, annual medical examination status, and the presence of chronic disease as confounding variables; and psychological resilience as the mediator. The model was estimated using the maximum likelihood method. The results indicated satisfactory overall model fit: χ² = 225.441, DF = 87, *P* < 0.001, and the other key indices also met the criteria for acceptance, detailed results are presented in Table 8. The final structural equation model plot is shown in Fig. 3.

**Table 8:** Indicators of model fit

Goodness-of-fit Metrics	χ^2^/DF	GFI	NFI	TLI	CFI	RMSEA
Model Results	2.591	0.924	0.910	0.930	0.942	0.066
Evaluation Criteria	[1,3]	>0.9	>0.9	>0.9	>0.9	<0.08

### Path relationship analysis

In the structural equation model, social support was significantly positively associated with psychological resilience (*β* = 0.662; *P* < 0.001). Social support was also positively associated with HPL (*β* = 0.241, *P* < 0.001), and psychological resilience was similarly associated with HPL (*β* = 0.577, *P* < 0.001). The standardized path coefficients between variables are shown in Table 9.

**Table 9:** Standardized path test results for SEM

Path	β	SE	*P*
Social Support—> Psychological Resilience	0.662	0.347	<0.001
Social Support—> HPL	0.241	0.125	<0.001
Psychological Resilience—> HPL	0.577	0.024	<0.001

### Assessment of indirect association

To further examine the significance of the hypothesized indirect pathway, the bootstrap method with 5000 resamples was applied to estimate the 95% *CI* [34]. The results indicated that the indirect association involving psychological resilience was significant, with a 95% *CI* of [0.495,0.929], *P* < 0.001. These findings suggest that psychological resilience serves as a significant intermediary in the observed relationship. The direct association between social support and HPL was also significant (95% *CI* [0.168, 0.714]; *P* < 0.001). Bootstrap testing indicated that psychological resilience partially mediated the relationship between social support and HPL, which accounted for 61.32% of the total effect. The detailed results are presented in Table 10.

**Table 10:** Bootstrap mediation effect test results

Association	Effect Estimate	95% CI			Proportion of Total Effect
		Lower	Upper	*P*	
Direct Association	0.432	0.168	0.714	*P*<0.001	38.68%
Indirect Association	0.685	0.495	0.929	*P*<0.001	61.32%
Total Association	1.117	0.884	1.373	*P*<0.001	

Direct association is social support → HPL; indirect association is social support → psychological resilience → HPL

## Discussion

This study constructed and validated a theoretical model in which psychological resilience significantly mediates the relationship between social support and HPL among perimenopausal women in China.

The mean HPL score in our sample indicated a moderate level of health-promoting behaviours, which is consistent with findings in comparable populations [35]. Among the HPLP subscales, the nutrition dimension received the highest ratings. This focus on dietary habits is likely driven by multiple contextual factors, including the growing availability of online nutritional information [36], the development of multidisciplinary menopause clinics offering dietary guidance in certain Chinese regions [37], and a broader cultural emphasis on the significance of food [38]. In contrast, the lowest scores were observed in the physical activity subscale, which may reflect both physiological barriers—such as an elevated risk of osteoporosis [39] and a high prevalence of pain symptoms [40] —and social structural constraints, including substantial family care responsibilities [41].

Multiple linear regression indicated that women who had undergone menopause or who had received regular health check-ups reported higher HPLP scores—a pattern aligned with prior research [35]. This association may be partly explained by heightened health awareness following menopause, a transition period marked by increased risks of cardiovascular disease [42] and osteoporosis [43], which could motivate the adoption of behaviours aimed at symptom management. Additionally, regular check-ups provide structured opportunities for professional health guidance, thereby reinforcing healthy behaviours [44]. Conversely, the presence of chronic diseases was associated with lower HPLP scores, which is consistent with findings from some adult populations [45]. Chronic conditions are frequently accompanied by psychological distress, such as anxiety and depression [46], which may undermine self-efficacy and impede the adoption of health-promoting behaviours. Nevertheless, the relationship between chronic disease and lifestyle remains complex and has not been consistently observed across studies, suggesting a need for further investigation [47]. Collectively, these findings underscore the importance of health awareness, access to professional support, and psychological well-being in shaping lifestyle behaviours during the menopausal transition.

Notably, social support and psychological resilience emerged as strong and consistent predictors of HPL in both the regression and SEM analyses.

The positive association between social support and HPL aligns with prior findings in pregnant women [48] and other populations [49], supporting the theoretical view that social resources facilitate the maintenance of healthy behaviours [21]. Moreover, from a sociopsychological perspective, social support, empowerment, and equitable relationships are crucial for promoting women’s mental health [50], complementing traditional biomedical approaches. In the context of perimenopause—a period marked by substantial physiological, psychological, and social transitions—social support may serve to buffer stress, enhance self-efficacy, and provide practical resources that facilitate healthier behavioural choices [51, 52].

Importantly, the SEM results further elucidated this relationship by demonstrating a significant mediating role of psychological resilience. The indirect effect accounted for approximately 61.32% of the total association between social support and HPL, indicating that a substantial portion of the influence of social support is channelled through enhanced adaptive psychological capacity. This pattern can be interpreted through the lens of the stress-moderation model [53]. In this framework, psychological resilience can be viewed as a dispositional factor that buffers the pathogenic effects of stress (e.g., perimenopausal challenges). Our results further specify that social support (an external resource) is a key antecedent that enhances this resilience, thereby indirectly promoting healthier behaviours—a pathway consistent with the model’s focus on vulnerability and protective factors. This interpretation is consistent with previous research linking lower resilience to more severe menopausal symptoms and poorer health-related adaptation [54], and higher resilience to more favourable health outcomes [55, 56].

Together, these findings integrate two prominent theoretical perspectives: the main-effect model of social support, which highlights the direct role of social support in providing resources and reinforcing healthy norms, and the buffering model, wherein support mitigates stress through enhancing adaptive capacities such as resilience. Our results suggest that in perimenopausal women, social support functions as both a direct resource for health behaviour and a buffer that operates substantially through psychological resilience.

In summary, social support is directly associated with healthier lifestyles among perimenopausal women, and psychological resilience appears to play a substantial intermediary role. These findings highlight the potential value of integrating both social and psychological resources into health promotion programs for this population. Interventions that strengthen social networks while also fostering resilience skills may offer a synergistic pathway to support lasting health behaviour changes during the menopausal transition.

### Strengths and limitations

Our study has two strengths. First, it takes a close look at the current state and factors that affect the HPLs of women in perimenopause, as well as the connections between these factors. These findings can provide a reference basis for researchers to carry out relevant intervention studies. Second, we collected data in this study through face-to-face interviews, which ensures the authenticity and reliability of the research results. However, this study has several limitations. First, the cross-sectional design precludes causal inference and longitudinal assessment. While SEM is used to explore potential pathways, the temporal and causal relationships need longitudinal verification. Second, convenience sampling from a single hospital likely resulted in the recruitment of more health-conscious women with better health care access, limiting the generalizability of the findings to the broader perimenopausal population, especially those with lower health engagement. Third, despite covariate control, unmeasured confounding remains possible. Methodologically, using subscale scores (instead of item-level indicators) for latent variables enhances parsimony but may not fully account for measurement error, potentially affecting estimate precision. Future studies should confirm our findings using a full measurement model, employ probability sampling across diverse settings, and adopt longitudinal or intervention designs to strengthen causal inference and generalizability.

### Practice implications

The findings of this study highlight the critical role of social support and psychological resilience in promoting a healthy lifestyle among perimenopausal women. On the basis of our findings, we propose the following recommendations for clinicians and the health care system to enhance perimenopausal women’s health management.

(1) Strengthen social support networks: Health care professionals can design and implement perimenopausal health education programs that include spouses or key family members [57, 58]. These programs aim to disseminate knowledge about perimenopause, helping family members understand its physiological and psychological characteristics, thereby reinforcing women’s support systems and encouraging the adoption of healthier lifestyle behaviours. Additionally, advocating for integrated support systems that involve families, communities, and health care institutions may help reduce stigma and improve the overall support environment.

(2) Improved psychological resilience support groups for perimenopausal women can be established through communities or health care institutions. Regular offline exchanges or online interactions can encourage experience sharing and emotional mutual aid, creating a sustainable peer support environment. This approach helps women perceive perimenopause as a natural life stage and strengthens positive adaptations. Offering individualized psychological support may further enhance resilience and confidence during this transition.

(3) Develop and promote accessible digital health resources: Given that many perimenopausal women seek health information online but often encounter materials that are difficult to comprehend [59], health care providers should develop and disseminate scientifically sound, easy-to-understand digital content on healthy lifestyles. Utilizing digital platforms to provide reliable information can broaden access to informational support and assist women in making informed health decisions [60].

(4) Raise self-care awareness and promote preventive health behaviours: The importance of regular physical examinations should be emphasized, such as encouraging perimenopausal women to undergo at least one check-up annually and enhancing communication with health care professionals during these visits. Women can also be taught self-examination methods, such as breast self-checks and menstrual observation, to improve their awareness of their health status. Strengthening health responsibility and self-care consciousness may motivate more consistent engagement in healthy behaviours.

## Conclusions

This study revealed that perimenopausal women exhibited a moderate level of health-promoting behaviours, with nutrition being relatively well managed but physical activity notably inadequate. The findings indicate that social support is associated with an HPL both directly and indirectly, where psychological resilience serves as a key mediator. These findings suggest that social support not only directly facilitates health behaviours but also buffers stress by strengthening psychological resilience as an adaptive resource. Thus, in promoting health among perimenopausal women, emphasis should be placed on the synergistic development of social support systems and psychological resilience.

## Data Availability

In order to safeguard the privacy of the research participants, the data from this study cannot be publicly shared. The datasets generated and analysed during the current study are available from the corresponding author upon reasonable request.

## Abbreviations

HPL: Health-Promoting Lifestyle
STRAW: Stages of Reproductive Aging Workshop
HPLP: Health-Promoting Lifestyle Profile
SSRS: Social Support Rating Scale
CD-RISC: Connor-Davidson Resilience Scale
SEM: Structural equation modelling
CFA: Confirmatory factor analysis
CFI: Comparative fit index
TLI: Tucker-Lewis index
RMSEA: Root mean square error of approximation
CR: Composite reliability
AVE: Average variance extracted

## References

[1] Harlow SD, Gass M, Hall JE, Lobo R, Maki P, Rebar RW, Sherman S, Sluss PM, de Villiers TJ. STRAW CG: Executive summary of the Stages of Reproductive Aging Workshop + 10: addressing the unfinished agenda of staging reproductive aging. *CLIMACTERIC* 2012;15(2):105–114. 10.3109/13697137.2011.650656 PMC3580996 22338612

[2] Weber MT, Rubin LH, Schroeder R, Steffenella T, Maki PM. Cognitive profiles in perimenopause: hormonal and menopausal symptom correlates. CLIMACTERIC. 2021;24(4):401–7. 33759672 10.1080/13697137.2021.1892626

[3] Blackson EA, Mccarthy C, Bell C, Ramirez S, Bazzano AN. Experiences of menopausal transition among populations exposed to chronic psychosocial stress in the united states: a scoping review. BMC WOMENS HEALTH. 2024;24(1):487. 10.1186/s12905-024-03329-z PMC11373288 39232712

[4] Kaplan V. Mental health States of housewives: an evaluation in terms of Self-perception and codependency. INT J MENT HEALTH AD. 2023;21(1):666–83. 10.1007/s11469-022-00910-1 PMC9447951 36091486

[5] Rautenberg TA, Ng S, Downes M. A cross-sectional study of symptoms and health-related quality of life in menopausal-aged women in China. BMC WOMENS HEALTH. 2023;23(1):563. 10.1186/s12905-023-02728-y PMC10621238 37915020

[6] Reshadat S, Tohidi M, Ghasemi M, Zangeneh A, Saeidi S, Teimouri R, Yigitcanlar T. Interrelationship between underprivileged neighborhoods and health promotion lifestyles: insights from Kermanshah, Iran. J PUBLIC HEALTH-HEID. 2020;28(6):693–702.

[7] Sadeghi R, Arefi Z, Shojaeizadeh D, Shaahmadi F. The impact of educational intervention based on pender’s health promotion model on healthy lifestyle in women of reproductive age in Iran. J Lifestyle Med. 2022;12(2):83–8. 36157888 10.15280/jlm.2022.12.2.83 PMC9490014

[8] Abdelaziz EM, Elsharkawy NB, Mohamed SM. Health promoting lifestyle behaviors and sleep quality among Saudi postmenopausal women. FRONT PUBLIC HEALTH. 2022;10:859819. 10.3389/fpubh.2022.859819 PMC9240311 35784250

[9] Masten AS, Obradović J. Competence and resilience in development. ANN NY ACAD SCI. 2006;1094(1):13–27. 17347338 10.1196/annals.1376.003

[10] Kuck MJ, Hogervorst E. Stress, depression, and anxiety: psychological complaints across menopausal stages. FRONT PSYCHIATRY. 2024;15:1323743. 10.3389/fpsyt.2024.1323743 PMC10917984 38455517

[11] Bhattarai M, Jin Y, Smedema SM, Cadel KR, Baniya M. The relationships among self-efficacy, social support, resilience, and subjective well-being in persons with spinal cord injuries. J ADV NURS. 2021;77(1):221–30. 33009842 10.1111/jan.14573

[12] McElhany K, Aggarwal S, Wood G, Beauchamp J. Protective and harmful social and psychological factors associated with mood and anxiety disorders in perimenopausal women: A narrative review. MATURITAS. 2024;190:108118. 10.1016/j.maturitas.2024.108118 39317031

[13] Coronado PJ, Oliva A, Fasero M, Piñel C, Herraiz MA, Pérez-López FR. Resilience and related factors in urban, mid-aged Spanish women. CLIMACTERIC. 2015;18(6):867–72. 26029984 10.3109/13697137.2015.1045483

[14] Süss H, Ehlert U. Psychological resilience during the perimenopause. MATURITAS. 2020;131:48–56. 31787147 10.1016/j.maturitas.2019.10.015

[15] Chedraui P, Pérez-López FR, Schwager G, Sánchez H, Aguirre W, Martínez N, Miranda O, Plaza MS, Astudillo C, Narváez J. Resilience and related factors during female Ecuadorian mid-life. MATURITAS. 2012;72(2):152–6. 22464231 10.1016/j.maturitas.2012.03.004

[16] Poreba-Chabros A, Mamcarz P, Jurek K. Social support as a moderator between the perception of the disease and stress level in lung cancer patients. ANN AGR ENV MED. 2020;27(4):630–5. 33356071 10.26444/aaem/123099

[17] Pala SC, Unsal A, Arslantas D, Ocal EE, Dagtekin G. Evaluation of depression, social support and quality of life in perimenopausal and postmenopausal women in semi-rural Turkey. Psychogeriatrics. 2022;22(5):679–87. 35778987 10.1111/psyg.12870

[18] Qin W. Health behavior changes after a diabetes diagnosis: the moderating role of social support. *BEHAV MED*. 2023;49(3):292-301. 10.1080/08964289.2022.2050670 PMC9519805 35350953

[19] Fontes A, Pereira CR, Menezes S, Soares A, Almeida P, Carvalho G, Arriaga P. Predictors of Health-Protective and helping behaviors during the Covid-19 pandemic: the role of social support and resilience. *PSYCHOL REP*. 2024;127(6):2736-2761. 10.1177/00332941221123777 PMC11528867 36036086

[20] Obara-Golebiowska M. Early Maladaptive Schemas, Emotion Regulation, Stress, Social Support, and Lifestyle Factors as Predictors of Eating Behaviors and Diet Quality: Evidence from a Large Community Sample. *NUTRIENTS* 2025;17(20):3188. 10.3390/nu17203188 PMC12566790 41156441

[21] Berkman LF, Glass T, Brissette I, Seeman TE. From social integration to health: Durkheim in the new millennium. SOC SCI MED. 2000;51(6):843–57. 10972429 10.1016/s0277-9536(00)00065-4

[22] Cong H, Juan M, Lei XY, Mejia A, Marcellin D, Zhong C. Psychological resilience and social support as determinants of HIV-related stress in older adults living with HIV in china: a moderated mediation analysis. BMC Public Health Process. 2025;25(1):3864. 10.1186/s12889-025-25159-w PMC12599025 41214654

[23] Choompunuch B, Lebkhao D, Suk-erb W, Matsuo H. Health-Promoting behaviors and their associations with Frailty, Depression, and social support in Thai Community-Dwelling older adults: A Cross-Sectional analysis. ANN GERIATR MED RES. 2025;29(3):393–402. 41025274 10.4235/agmr.25.0080 PMC12489605

[24] Cohen S, Wills TA. Stress, social support, and the buffering hypothesis. PSYCHOL BULL. 1985;98(2):310. 3901065

[25] Gong YF, Zhou YH, Zhou D, Feng A, Zhang BL, Wang JM, Zhao L. Associations between social Support, health literacy and psychological resilience to Self-Management behaviours in liver transplant Recipients-A structural equation model. J CLIN NURS. 2025;34(10):4366–76. 40084794 10.1111/jocn.17697

[26] Memon MA, Ting H, Hwa CJ, Ramayah T, Cham TH. Sample Size for Survey Research: Review and Recommendations. JOURNAL OF APPLIED STUCTURAL EQUATION MODELING. 2020;4(2):i-xx.

[27] Lee H, Lee BG, La IS. Differential patterns of lifestyle behaviors among low- and high-income postmenopausal women in korea: a latent class analysis. BMC WOMENS HEALTH. 2023;23(1):617. 10.1186/s12905-023-02767-5 PMC10657161 37980479

[28] Walker SN, Sechrist KR, Pender NJ. The Health-Promoting lifestyle profile: development and psychometric characteristics. NURS RES. 1987;36(2):76–81. 3644262

[29] Cao WJ, Chen CS, Hua Y, Li YM, Xu YY, Hua QZ. Factor analysis of a health-promoting lifestyle profile (HPLP): application to older adults in Mainland China. ARCH GERONTOL GERIAT. 2012;55(3):632–8. 10.1016/j.archger.2012.07.003 22854282

[30] SY Xiao. Theoretical basis and research applications of the social support rating scale [in Chinese]. J CLIN PSYCHIAT. 1994;4(02):98–100.

[31] Connor KM, Davidson JRT. Development of a new resilience scale: the Connor-Davidson resilience scale (CD-RISC). DEPRESS ANXIETY. 2003;18(2):76–82. 12964174 10.1002/da.10113

[32] Yu XN, Zhang JX. Factor analysis and psychometric evaluation of the Connor-Davidson resilience scale (CD-RISC) with Chinese people. SOC BEHAV PERSONAL. 2007;35(1):19–30.

[33] Preacher KJ, Hayes AF. Asymptotic and resampling strategies for assessing and comparing indirect effects in multiple mediator models. BEHAV RES METHODS. 2008;40(3):879–91. 18697684 10.3758/brm.40.3.879

[34] Hayes AF. Introduction to mediation, moderation, and conditional process analysis: A regression-based approach. 2nd ed. New York: Guilford Press; 2017.

[35] Ashgar RI, Krishnasamy T. Health promotion behaviors and psychosocial factors among Middle-Aged women in Saudi Arabia. SAGE OPEN NURS. 2023;9:23779608231187263. 10.1177/23779608231187263 PMC10336765 37448970

[36] Lu J, Li KF, Zheng XL, Liu R, Chen M, Xian JY, Tu SH, Xie LL. Prevalence of menopausal symptoms and attitudes towards menopausal hormone therapy in women aged 40–60 years: a cross-sectional study. BMC WOMENS HEALTH. 2023;23(1):472. 10.1186/s12905-023-02621-8 PMC10476428 37667324

[37] Kornstein SG, Pinkerton JV, Pace DT, Singer AJ, Kingsberg SA, Ellis LE, Ashley P, Klein W. Multidisciplinary Management of Menopause: Symposium Proceedings. *J WOMENS HEALTH* 2022;31(8):1071–1078. 10.1089/jwh.2022.0175 35980244

[38] Li XM, Li XD, Liao YL, Zhu GH, Yu GX. Analysis of residents’ food safety satisfaction from the perspective of income heterogeneity. SCI REP-UK . 2021;11(1):6666. 10.1038/s41598-021-85384-2 PMC7988056 33758212

[39] Fistarol M, Rezende CR, Campos A, Kakehasi AM, Geber S. Time since menopause, but not age, is associated with increased risk of osteoporosis. CLIMACTERIC. 2019;22(5):523–6. 31280605 10.1080/13697137.2019.1634046

[40] Gibson CJ, Li YM, Bertenthal D, Huang AJ, Seal KH. Menopause symptoms and chronic pain in a National sample of midlife women veterans. MENOPAUSE. 2019;26(7):708–13. 30839364 10.1097/GME.0000000000001312

[41] Qian Y, Qian ZC. Work, Family, and gendered happiness among married people in urban China. SOC INDIC RES. 2015;121(1):61–74.

[42] Chair SY, Lo S, Cheung HY, Sit J, Wang Q, Zou HJ. Vasomotor symptoms, cardiovascular risk factors, and cardiovascular disease risk among Chinese postmenopausal women in Hong Kong. WOMEN HEALTH. 2022;62(7):621–32. 35876176 10.1080/03630242.2022.2100034

[43] Abu Khurmah MH, Alkhatatbeh MJ, Alshogran OY, Alarda HM. Prevalence and risk factors of osteopenia and osteoporosis among postmenopausal women: A cross-sectional study from Jordan. PUBLIC HEALTH NURS. 2024;41(5):996–1005. 39037197 10.1111/phn.13379

[44] Neves-e-Castro M, Birkhauser M, Samsioe G, Lambrinoudaki I, Palacios S, Borrego RS, Llaneza P, Ceausu I, Depypere H, Erel CT. EMAS position statement: the ten point guide to the integral management of menopausal health. MATURITAS. 2015;81(1):88–92. 25757366 10.1016/j.maturitas.2015.02.003

[45] Lin YH, Chu LL. The health promotion lifestyle of metabolic syndrome individuals with a diet and exercise programme. INT J NURS PRACT. 2014;20(2):142–8. 24713010 10.1111/ijn.12149

[46] Gaggero A, Gil J, Jiménez-Rubio D, Zucchelli E. Sick and depressed? The causal impact of a diabetes diagnosis on depression. HEALTH ECON REV. 2023;13(1):38. 10.1186/s13561-023-00451-w PMC10316538 37395821

[47] Enjezab B, Farajzadegan Z, Taleghani F, Aflatoonian A, Morowatisharifabad MA. Health promoting behaviors in a population-based sample of middle-aged women and its relevant factors in Yazd, Iran. INT J Prev MED. 2012;3(Suppl1):S191. 22826765 PMC3399308

[48] Bahabadi FJ, Estebsari F, Rohani C, Kandi Z, Sefidkar R, Mostafaei D. Predictors of health-Promoting lifestyle in pregnant women based on pender’s health promotion model. INT J WOMENS HEALTH. 2020;12:71–7. 32158276 10.2147/IJWH.S235169 PMC7047988

[49] Aqtash S, Van Servellen G. Determinants of Health-Promoting lifestyle behaviors among Arab immigrants from the region of the levant. RES NURS HEALTH. 2013;36(5):466–77. 24037811 10.1002/nur.21555

[50] Kaplan V. Gender sensitive psychiatry and feminist therapy. Kıbrıs Türk Psikiyatri Ve Psikoloji Dergisi. 2021;3(3):211–6.

[51] Aloufi B, Hassanien NS. The association of menopausal symptoms and social support among Saudi women at primary health care centers in Taif, Saudi Arabia. CUREUS J MED Sci. 2022;14(6):e26122. 10.7759/cureus.26122 PMC9298676 35875302

[52] Denche-Zamorano A, García-Paniagua R, Pastor-Cisneros R, Pereira-Payo D, Gómez JP. Influence of physical activity level and perceived social support on mental health and psychological distress in women with menopause problems. PSYCHOL HEALTH MED. 2024;29(8):1493–511. 38712645 10.1080/13548506.2024.2347522

[53] Wiebe DJ, Smith TW. Personality and health: Progress and problems in psychosomatics. In: Hogan R, Johnson JA, Briggs SR, eds. Handbook of Personality Psychology. Academic Press; 1997. pp. 891-918.

[54] Zhao D, Liu C, Feng X, Hou F, Xu X, Li P. Menopausal symptoms in different substages of perimenopause and their relationships with social support and resilience. MENOPAUSE. 2019;26(3):233–9. 30252803 10.1097/GME.0000000000001208

[55] Chretien A, Hayotte M, Vuillemin A, Longueville FD. Resilience profiles of elite athletes and their associations with health-related behaviors, well-being, and performance: A latent profile analysis. PSYCHOL SPORT EXERC. 2024;74:102689. 10.1016/j.psychsport.2024.102689 38901549

[56] Manning LK, Carr DC, Kail BL. Do higher levels of resilience buffer the deleterious impact of chronic illness on disability in later life? Gerontologist. 2016;56(3):514–24. 25063353 10.1093/geront/gnu068 PMC4873762

[57] Chiang YC, Lee HC, Chu TL, Wu CL, Hsiao YC. The relationship between spiritual health, health-promoting behaviors, depression and resilience: A longitudinal study of new nurses. NURSE EDUC PRACT. 2021;56:103219. 10.1016/j.nepr.2021.103219 34628178

[58] Bahri N, Yoshany N, Morowatisharifabad MA, Noghabi AD, Sajjadi M. The effects of menopausal health training for spouses on women’s quality of life during menopause transitional period. MENOPAUSE. 2016;23(2):183–8. 26783984 10.1097/GME.0000000000000588

[59] Charbonneau DH. Readability of menopause web sites: A Cross-Sectional study. J WOMEN AGING. 2012;24(4):280–91. 23098043 10.1080/08952841.2012.708574

[60] Cowell AC, Gilmour A, Atkinson D. Support Mechanisms for Women during Menopause: Perspectives from Social and Professional Structures. *WOMEN* 2024;4(1):53–72.

